# Mechanism of Snhg8/miR-384/Hoxa13/FAM3A axis regulating neuronal apoptosis in ischemic mice model

**DOI:** 10.1038/s41419-019-1631-0

**Published:** 2019-06-05

**Authors:** Jie Liu, Ping An, Yixue Xue, Dongfang Che, Xiaobai Liu, Jian Zheng, Yunhui Liu, Chunqing Yang, Zhen Li, Bo Yu

**Affiliations:** 1 Department of Neurosurgery, Shengjing Hospital of China Medical University, Shenyang, China; 2 Key Laboratory of Neuro-oncology in Liaoning Province, Shenyang, China; 3 Liaoning Clinical Medical Research Center in Nervous System Disease, Shenyang, China; 4 Department of Neurobiology, College of Basic Medicine, China Medical University, Shenyang, China; 5 Key Laboratory of Cell Biology, Ministry of Public Health of China, China Medical University, Shenyang, China; 6 Key Laboratory of Medical Cell Biology, Ministry of Education of China, China Medical University, 110122 Shenyang, China

**Keywords:** Long non-coding RNAs, miRNAs, Cell death in the nervous system

## Abstract

Long noncoding RNAs, a subgroup of noncoding RNAs, are implicated in ischemic brain injury. The expression levels of Snhg8, miR-384, Hoxa13, and FAM3A were measured in chronic cerebral ischemia-induced HT22 cells and hippocampal tissues. The role of the Snhg8/miR-384/Hoxa13/FAM3A axis was evaluated in chronic cerebral ischemia models in vivo and in vitro. In this study, we found that Snhg8 and Hoxa13 were downregulated, while miR-384 was upregulated in chronic cerebral ischemia-induced HT22 cells and hippocampal tissues. Overexpression of Snhg8 and Hoxa13, and silencing of miR-384, all inhibited chronic cerebral ischemia-induced apoptosis of HT22 cells. Moreover, Snhg8 bound to miR-384 in a sequence-dependent manner and there was a reciprocal repression between Snhg8 and miR-384. Besides, overexpression of miR-384 impaired Hoxa13 expression by targeting its 3′UTR and regulated chronic cerebral ischemia-induced neuronal apoptosis. Hoxa13 bound to the promoter of FAM3A and enhanced its promotor activity, which regulated chronic cerebral ischemia-induced neuronal apoptosis. Remarkably, the in vivo experiments demonstrated that Snhg8 overexpression combined with miR-384 knockdown led to an anti-apoptosis effect. These results reveal that the Snhg8/miR-384/Hoxa13/FAM3A axis plays a critical role in the regulation of chronic cerebral ischemia-induced neuronal apoptosis.

## Introduction

Chronic cerebral ischemia (CCI) causes chronic ischemic neurological damage, which is involved in the development of dementia, such as Alzheimer’s disease (AD) and vascular dementia (VaD)^1^. CCI contributes to persistent and progressive cognitive impairment and neuronal damage^2^, in which hippocampal neurons are particularly severely damaged^3^. Therefore, the research on the mechanism of chronic cerebral ischemia may provide a potential innovative approach for CCI therapy.

The bilateral common carotid artery stenosis (BCAS) model is currently known as the most promising animal model of chronic cerebral ischemia^4^. However, its main drawback is an acute drop of cerebral blood flow (CBF) and gradual CBF recovery due to compensatory mechanism^5^. Therefore, in this experiment, we applied a novel chronic cerebral ischemia model: the chronic cerebral ischemia model induced by ameroid constrictors (ACs). ACs, which consists of a titanium casing surrounding a hygroscopic casein material with an internal lumen, are applied to the bilateral common carotid arteries of the mice. Then the casein component gradually absorbs water and consequently swells, leading to predictable narrowing of arterial lumen it encases, which eventually causes relatively slow and steady decrease in CBF. Compared with the traditional chronic cerebral ischemia model, the AC model simulates the hemodynamic changes in patients with chronic cerebral ischemia better^6,7^.

Long non-coding RNAs (lncRNAs), are defined as transcripts longer than 200 nucleotides that are not translated into proteins. Recent studies show that more than half of lncRNAs are expressed in brain tissue and regulate many physiological and pathological activities of the central nervous system^8^. For example, lncRNA MALAT1 regulates retinal neurodegeneration through CREB signaling^9^. LncRNA CRNDE expression is upregulated in glioma stem cells, overexpression of CRNDE inhibits apoptosis of glioma stem cells^10^. Small nucleolar RNA host gene 8 (Snhg8) is upregulated in human gastric cancer cells, and knockdown of Snhg8 inhibits cell growth^11^. However, the role of Snhg8 in CCI has not been reported.

It has been verified that miRNAs play vital roles in the regulation of gene expression and cell function, and their involvement in cell apoptosis has been widely reported. Researchers found that miRNA-27a regulated cardiomyocyte apoptosis by targeting interleukin-10-mediated pathway^12^. MiR-29 can regulate the apoptosis of H69 cells by negatively regulating Mcl-1^13^. MiR-384 is involved in the metabolic regulation of nerve cells in central nervous system^14^; meanwhile earlier study found abnormal expression of miR-384 in diabetic cardiomyopathy^15^, therefore we speculated that miR-384 may be involved in cell metabolism. Bioinformatics Software (miRDB) reveals that Snhg8 harbors a binding site of miR-384. However, the function of miR-384 in CCI-induced neuronal apoptosis remains unknown.

Hoxa13, which belongs to Homeobox gene family, participates in embryonic development, cell proliferation, differentiation, migration, and apoptosis^16^. Hoxa13 expression is upregulated in human gliomas and inhibits glioma cell apoptosis by activating Wnt and TGF-β-signaling pathway^17^. Besides, Hoxa13 regulates cell apoptosis and then influences limb morphogenesis in mice by directly targeting Aldh1a2^18^. In addition, using TargetScan and miRanda bioinformatics software analysis, a binding site was identified between miR-384 and Hoxa13. However, the expression and role of Hoxa13 in CCI have not been reported.

FAM3A, a subgroup of family with sequence similarity 3 (FAM3) gene family, participates in regulation of neuronal apoptosis. For instance, FAM3A protects against glutamate-induced toxicity by preserving calcium homeostasis in PC12 cells^19^. By scanning the promoter sequence of FAM3A, a putative binding site of Hoxa13 was found.

In this study, we first detected the expression of Snhg8, miR-384, and Hoxa13 in CCI-induced HT22 cells and hippocampal tissues. The interactions among these factors were further explored and their possible mechanism of action on CCI-induced neuronal apoptosis was clearly demonstrated, which was ultimately to identify a potential new molecular target for the treatment of CCI.

## Results

### Snhg8 and Hoxa13 expression were downregulated, miR-384 expression was upregulated in CCI-induced hippocampal neurons in vivo and in vitro

**Fig. 1:**
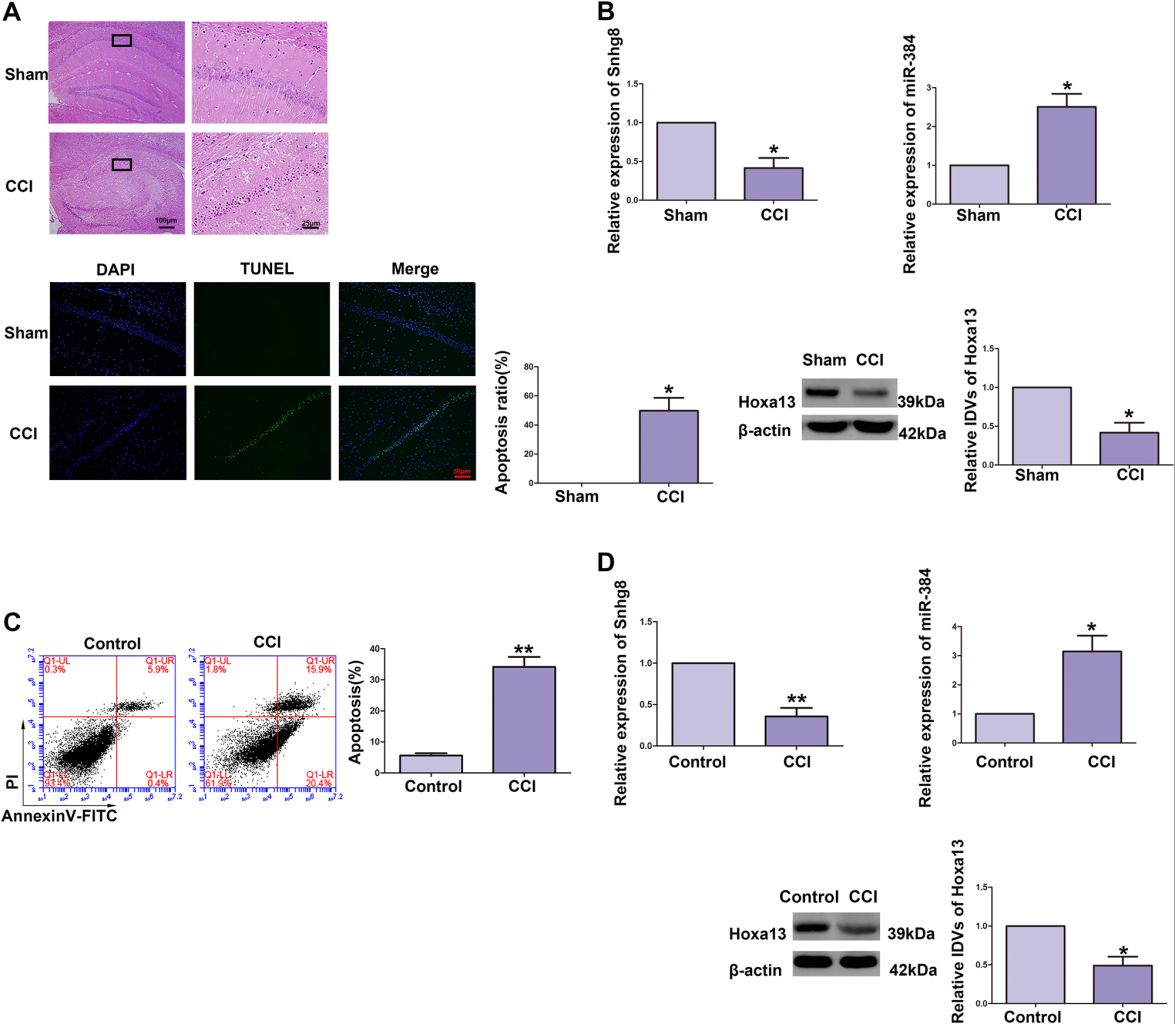
Effect of chronic cerebral ischemia (CCI) to neuronal apoptosis and relative expression levels of Snhg8, miR-384, and Hoxa13. **a** H&E-stained (upper part, ×100 or ×400, scale bar = 100 or 25 μm) and terminal deoxynucleotidyl transferase (TdT)-mediated dUTP-biotin nick end labeling (TUNEL) assay (lower part, ×200, scale bar = 50 μm) sections of the hippocampus CA1 region of Sham and CCI group (*n* = 4, each group). Data are represented as mean ± SD, *P < 0.05 vs. Sham group. **b** Quantitative real-time PCR and western blot (WB) were used to detect the relative expression levels of Snhg8 (upper left part, *n* = 5, each group), miR-384 (upper right part, *n* = 5, each group), and Hoxa13 (lower right part, *n* = 5, each group). Data are represented as mean ± SD, *P < 0.05 vs. Sham group. **c** Flow cytometry analysis of CCI-induced HT22 cells (*n* = 5, each group). Data are represented as mean ± SD, **P < 0.01 vs. Control group. **d** The expression levels of Snhg8 RNA (upper left part, *n* = 5, each group), miR-384 RNA (upper right part, *n* = 6, each group), and Hoxa13 protein (lower part, *n* = 5, each group) in CCI-induced HT22 cells. Data are represented as mean ± SD, *P < 0.05 vs. Control group, **P < 0.01 vs. Control group

To test the expression levels of Snhg8, miR-384, and Hoxa13 in CCI models in vivo and in vitro, RNA and protein samples were extracted from CCI-induced hippocampal tissues (Fig. 1a, b) and HT22 cells (Fig. 1c, d). Figure 1a is the example of H&E-stained (upper part, ×100 or ×400, scale bar = 100 or 25 μm) and TUNEL assay (lower part, ×200, scale bar = 50 μm) sections of the hippocampus CA1 region of Sham and CCI groups. As the H&E-stained sections shown, Hippocampal neuron atrophy, nuclear pyknosis in hippocampal CA1 region were detected. As the TUNEL assay shown, the nucleus was blue, and the TUNEL-positive cells (apoptotic cells) appeared green. Apoptosis rate of CCI group upregulated significantly (apoptosis ratio = TUNEL-positive neurons in CA1 area/total neurons number in CA1 area). As shown in Fig. 1b, quantitative real-time PCR (qRT-PCR) and western blot (WB) were used to detect endogenous expression of Snhg8 RNA (upper left part), miR-384 RNA (upper right part), and Hoxa13 protein (lower right part) in CCI-induced hippocampal neurons. Compared with Sham group, the expression of Snhg8 in CCI group was downregulated; the expression level of miR-384 was upregulated; the expression of Hoxa13 protein was downregulated. As shown in Fig. 1c, flow cytometry was used to detect the apoptosis ratio of HT22 cells induced by CCI. Compared with Control group, the apoptosis rate of neurons were upregulated in CCI groups. The endogenous expression of Snhg8 (upper left), miR-384 (upper right), and Hoxa13 (lower right) in CCI-induced HT22 cells was detected (Fig. 1d). Compared with the Control groups, the expression of Snhg8 was downregulated; the expression of miR-384 was upregulated; the expression of Hoxa13 protein was downregulated in the CCI groups.

### Snhg8 protected CCI-induced neuronal apoptosis, while miR-384 exacerbated CCI-induced neuronal apoptosis

**Fig. 2:**
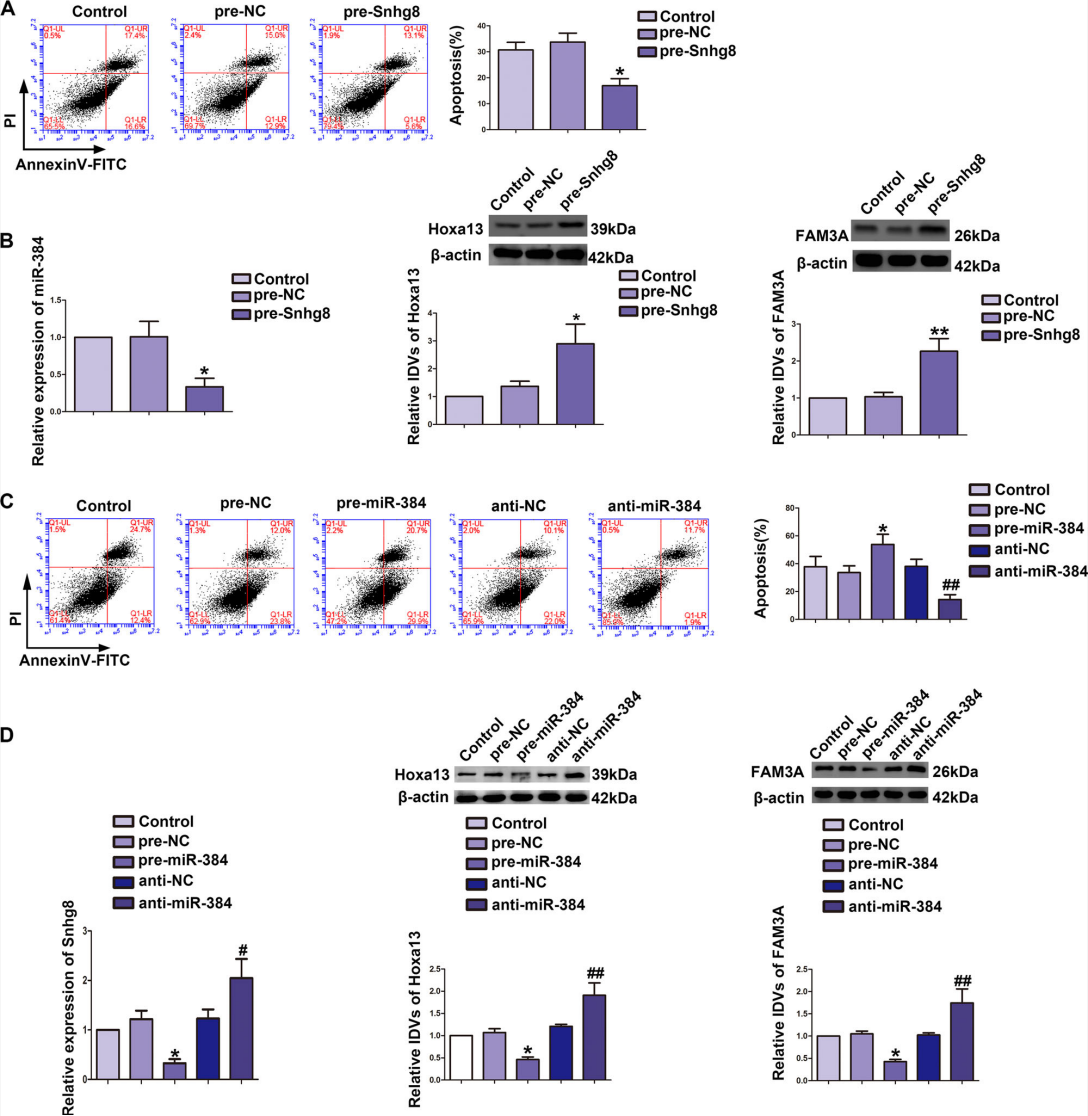
Snhg8 protected chronic cerebral ischemia (CCI)-induced neuronal apoptosis, while miR-384 exacerbated CCI-induced neuronal apoptosis. **a** Flow cytometry analysis of CCI-induced HT22 cells with overexpression of Snhg8 (*n* = 5, each group). Data are represented as mean ± SD, *P < 0.05 vs. Pre-NC group. **b** Expression analysis of miR-384 RNA (left part, *n* = 5, each group), Hoxa13 protein (middle part, *n* = 5, each group), and FAM3A protein (right part, *n* = 5, each group) in CCI-induced HT22 cells. Data are represented as mean ± SD, *P < 0.05 vs. Pre-NC group, **P < 0.01 vs. Pre-NC group. **c** Flow cytometry analysis of CCI-induced HT22 cells with transfection of miR-384 (*n* = 4, each group). Data are represented as mean ± SD, *P < 0.05 vs. Pre-NC group, ^##^*P* < 0.01 vs. anti-NC group. **d** Expression analysis of Snhg8 RNA (left part, *n* = 5, each group), Hoxa13 protein (middle part, *n* = 4, each group) and FAM3A protein (right part, *n* = 6, each group) in CCI-induced HT22 cells with transfection of miR-384. Data are represented as mean ± SD, *P < 0.05 vs. Pre-NC group, ^#^*P* < 0.05 vs. anti-NC group, ^##^*P* < 0.01 vs. anti-NC group

To determine the function of Snhg8 and miR-384 in CCI-induced neuronal apoptosis, CCI-induced HT22 cells were transfected with pcDNA3.1-Snhg8 and miR-384 antagomir, respectively. Apoptosis rate of pre-Snhg8 groups was significantly downregulated (Fig. 2a). Furthermore, reduced expression of miR-384 (Fig. 2b, left part) RNA and elevated expression levels of Hoxa13 (Fig. 2b, middle part) and FAM3A (Fig. 2b, right part) protein were detected. In contrast, apoptosis rate of CCI-induced HT22 cells was increased in pre-miR-384 groups (Fig. 2c) and decreased in anti-miR-384 groups (Fig. 2c). Furthermore, reduced expression of Snhg8 RNA (Fig. 2d, left part), Hoxa13 protein (Fig. 2d, middle part), and FAM3A (Fig. 2d, right part) protein were detected in pre-miR-384 groups; oppositely, increased expression of Snhg8 RNA (Fig. 2d, left part), Hoxa13 protein (Fig. 2d, middle part), and FAM3A protein (Fig. 2d, right part) were detected in anti-miR-384 groups.

### miR-384 targeted Snhg8 and reversed the Snhg8-mediated attenuation of CCI-induced neuronal apoptosis

**Fig. 3:**
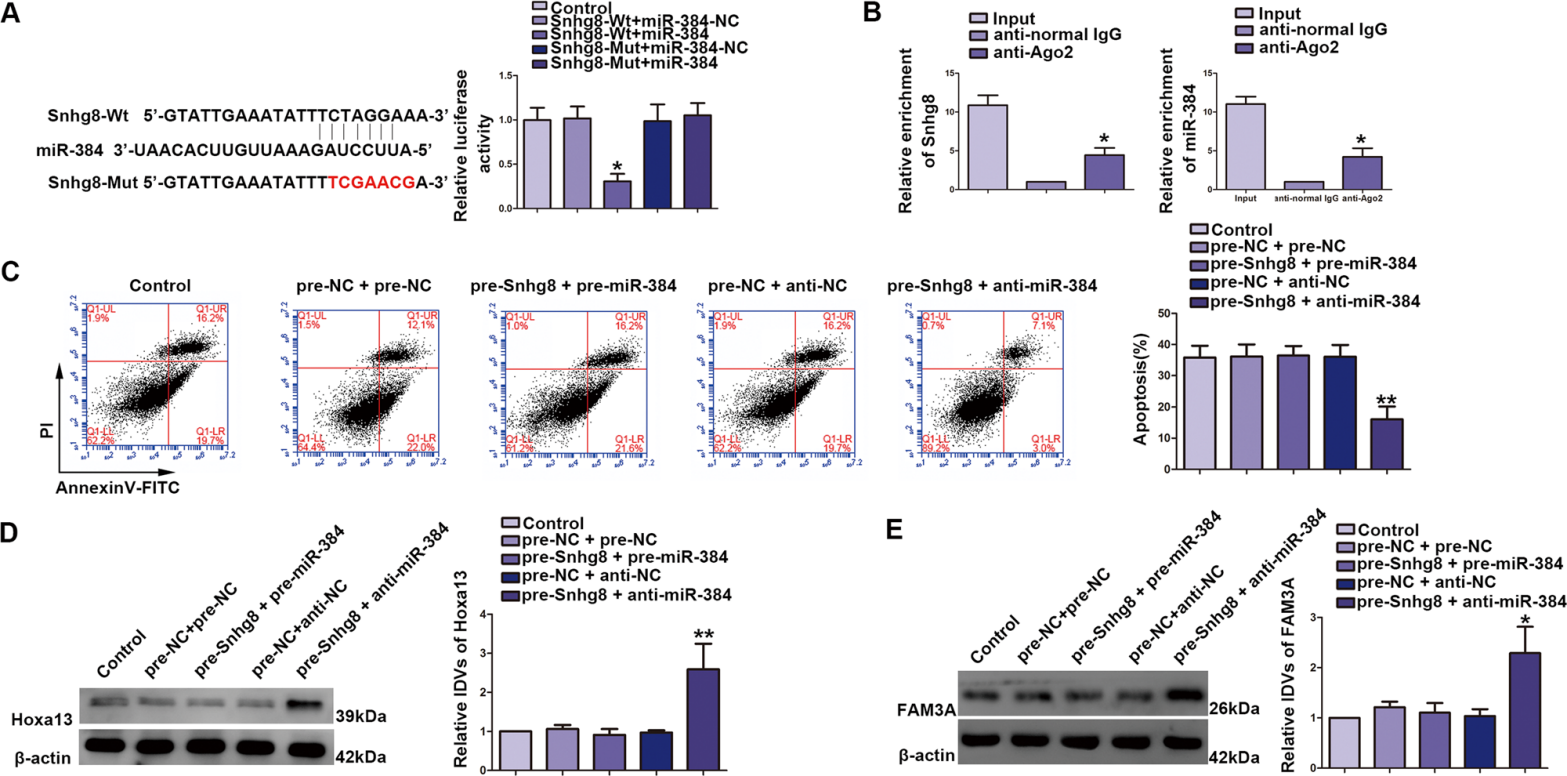
miR-384-targeted Snhg8 and reversed the Snhg8-mediated attenuation of chronic cerebral ischemia (CCI)-induced neuronal apoptosis. **a** The predicted miR-384-binding site in Snhg8 (Snhg8-Wt) and the designed mutant sequence (Snhg8-Mut) were indicated. Luciferase reporter assay of HT22 cells cotransfected with Snhg8-Wt or Snhg8-Mut and miR-384 or the miR-384-NC (data are presented as the mean ± SD (*n* = 4, each group). Data are displayed as mean ± SD, *P < 0.05 vs. Snhg8-Wt+miR-384-NC group. **b** miR-384 was identified in Snhg8-RISC complex. Relative expression levels of Snhg8 (*n* = 4, each group) and miR-384 were determined by qRT PCR (*n* = 4, each group). Data are displayed as mean ± SD, *P < 0.05 vs. anti-normal IgG. **c** Flow cytometry analysis of CCI-induced HT22 cells cotransfected with Snhg8 and miR-384 (*n* = 4, each group). Data are displayed as mean ± SD, **P < 0.01 vs. pre-NC+anti-NC group. **d** Hoxa13 protein levels in CCI-induced HT22 cells cotransfected with Snhg8 and miR-384 (*n* = 4, each group). Data are displayed as mean ± SD, **P < 0.01 vs. pre-NC+anti-NC group. **e** FAM3A protein levels in CCI-induced HT22 cells cotransfected with Snhg8 and miR-384 (*n* = 4, each group). Data are displayed as mean ± SD, **P < 0.01 vs. pre-NC+anti-NC group

To determine the mechanism of Snhg8 and miR-384 regulating CCI-induced neuronal apoptosis, CCI-induced HT22 cells were cotransfected with pcDNA3.1-Snhg8+miR-384 agomir and pcDNA3.1-Snhg8+agomir, respectively. Bioinformatics database (miRDB) illustrate that there was a putative-binding site between Snhg8 and miR-384 (Fig. 3a). We verified it by dual-luciferase reporter assay. Luciferase activity was significantly reduced in the Snhg8-Wt+miR-384 groups (Fig. 3a). In addition, RNA-binding protein immunoprecipitation (RIP) experiment was performed to determine whether Snhg8 and miR-384 were in a RNA-induced silencing complex (RISC). Snhg8 and miR-384 were both enriched in anti-Ago groups (Fig. 3b). Furthermore, overexpression of miR-384 reversed Snhg8-mediated attenuation of CCI-induced neuronal apoptosis. Compared with pre-Snhg8-NC+anti-miR-384-NC groups, apoptosis significantly decreased in pre-Snhg8+anti-miR-384 groups (Fig. 3c). As shown in Fig. 3d, compared with the pre-Snhg8-NC+anti-miR-384-NC groups, expression of Hoxa13 protein was significantly upregulated in pre-Snhg8+anti-miR-384 groups (left part); likewise, expression of FAM3A protein was significantly increased in pre-Snhg8+anti-miR-384 group (right part).

### Hoxa13 attenuated CCI-induced neuronal apoptosis by binding to the promotor of FAM3A, and overexpression of FAM3A inhibited CCI-induced neuronal apoptosis

**Fig. 4:**
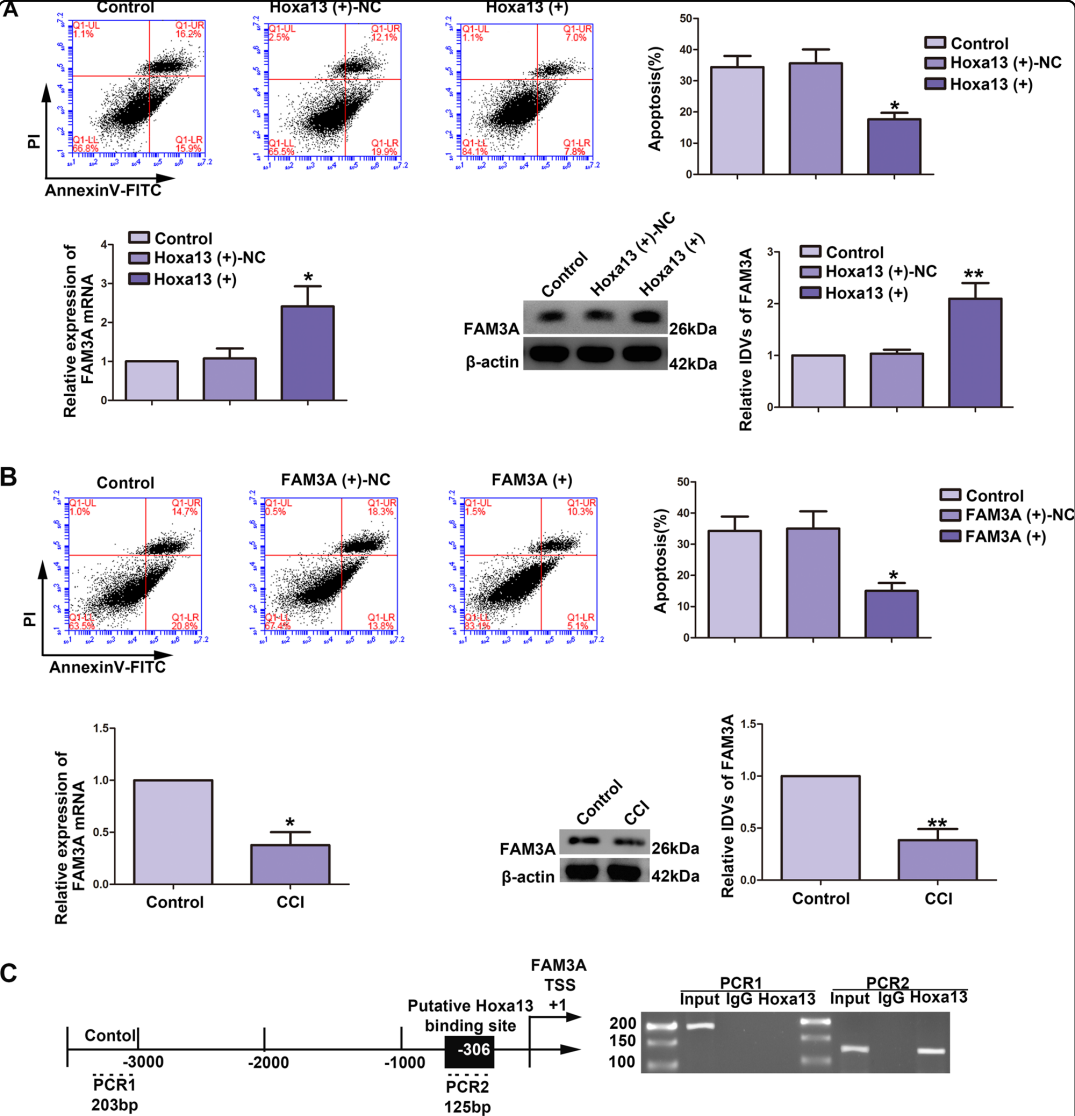
Overexpression of Hoxa13 and overexpression of FAM3A inhibited chronic cerebral ischemia (CCI) induced neuronal apoptosis. **a** Flow cytometry analysis of CCI-induced HT22 cells with overexpression of Hoxa13 (upper part, *n* = 4, each group). Expression analysis of FAM3A RNA (lower left part, *n* = 5, each group) and FAM3A protein (lower right part, *n* = 5, each group) in CCI-induced HT22 cells transfected with Hoxa13. Data are represented as mean ± SD, *P < 0.05 vs. Hoxa13 (+)-NC group, **P < 0.01 vs. Hoxa13 (+)-NC group. **b** Flow cytometry analysis of chronic cerebral ischemia CCI-induced HT22 cells with overexpression of FAM3A (upper part, *n* = 3, each group). Expression analysis of FAM3A RNA (lower left part, *n* = 4, each group) and FAM3A protein (lower right part, *n* = 6, each group) in CCI-induced HT22 cells. Data are represented as mean ± SD, *P < 0.05 vs. FAM3A (+)-NC group, *P < 0.05 vs. Control group, **P < 0.01 vs. Control group. **c** Putative FAM3A-binding sites are indicated. Immunoprecipitated DNA was amplified by PCR. Normal rabbit IgG was used as a negative control

Having confirmed that Hoxa13 was upregulated in hippocampal cells and tissues, effects of Hoxa13 on CCI-induced neuronal apoptosis were investigated. Apoptosis ratio of CCI-induced HT22 cells was decreased in Hoxa13 (+) group (Fig. 4a, upper part). FAM3A expression were upregulated both for RNA levels (Fig. 4a, left part) and protein levels (Fig. 4a, right part). Similarly, apoptosis ratio of CCI-induced HT22 cells was decreased in FAM3A (+) group (Fig. 4b, upper part). Additionally, reduced expression of FAM3A RNA (Fig. 4b, left part) and protein levels (Fig. 4b, right part) were detected. Biological information software (JASPA) shows that there is a binding site in the promoter of FAM3A for Hoxa13, predicted the promoter sequence of Hoxa13 and transcription start sites (TSSs) at the same time. By analyzing DNA sequences from 1000 bp region upstream to 200 bp region downstream of TSS, we confirmed the potential binding site (Fig. 4c). Meanwhile, we verified it via ChIP assay. Hoxa13 directly bound to the promoter region of FAM3A in HT22 cells, while in the corresponding negative control group, there was no combination between Hoxa13 and the control region (Fig. 4c).

### Overexpression of miR-384 impaired Hoxa13-induced attenuation of CCI-induced neuronal apoptosis by targeting 3´-UTR of Hoxa13

**Fig. 5:**
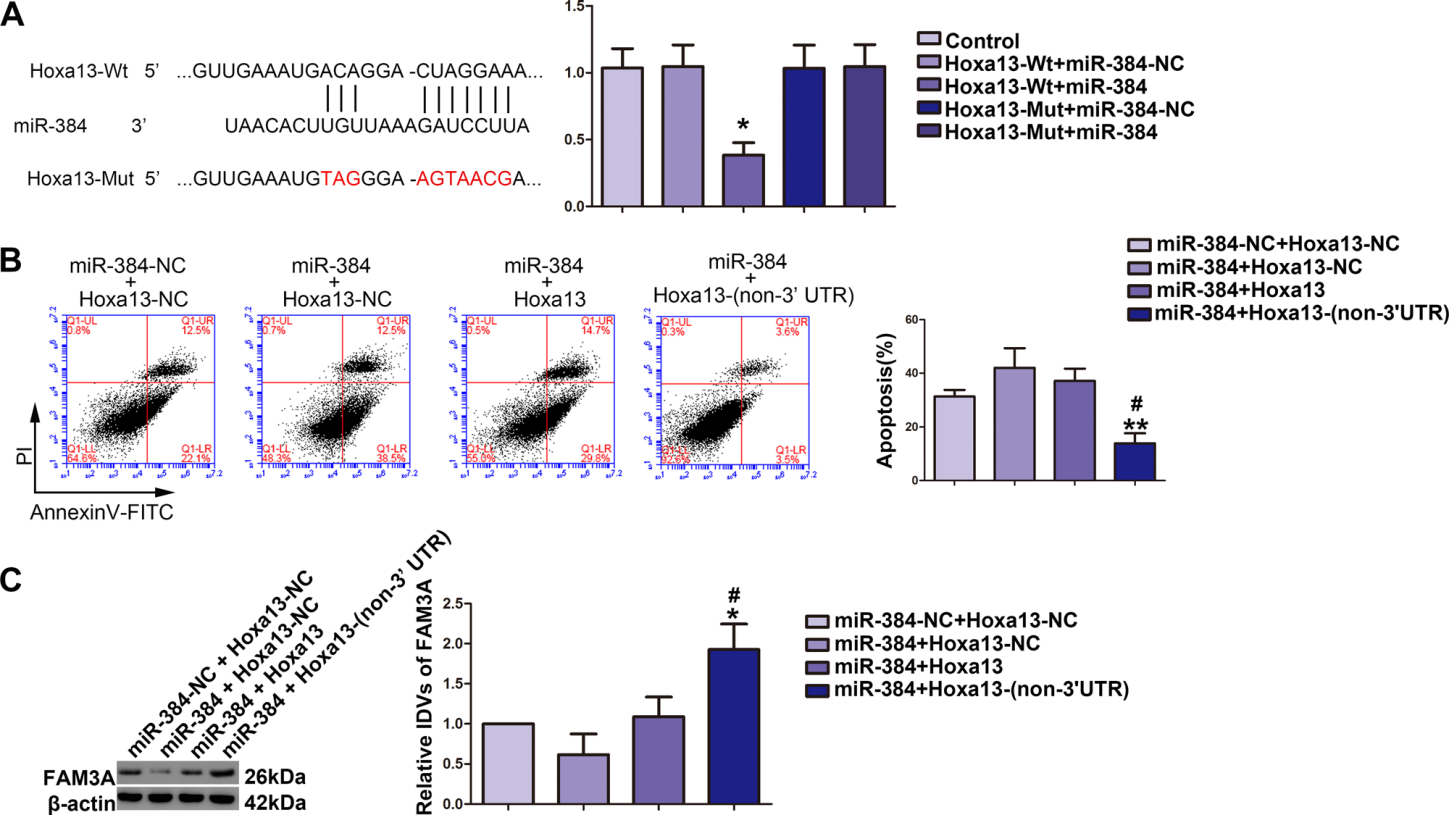
Overexpression of miR-384 impaired Hoxa13-induced attenuation of chronic cerebral ischemia (CCI)-induced neuronal apoptosis by targeting 3´-UTR of Hoxa13. **a** The predicted miR-384-binding site in Hoxa13 and the designed mutant sequences were indicated. Dual-luciferase report assay of HT22 cells cotransfected with Hoxa13-Wt and miR-384-NC or Hoxa13-Wt and miR-384, and Hoxa13-Mut and miR-384-NC or Hoxa13-Mut and miR-384 (*n* = 4, each group). Data are presented as mean ± SD, *P < 0.05 vs. Hoxa13-Wt+miR-384-NC group. **b** Flow cytometry analysis of CCI-induced HT22 cells cotransfected with miR-384 and Hoxa13/Hoxa13- (non-3´UTR) (*n* = 4, each group). Data are presented as mean ± SD, **P < 0.01 vs. miR-384+Hoxa13-NC group, ^#^*P* < 0.05 vs. miR-384+Hoxa13 group. **c** FAM3A protein levels in CCI-induced HT22 cells cotransfected with miR-384 and Hoxa13/Hoxa13-(non-3´UTR) (*n* = 4, each group). Data are presented as mean ± SD, *P < 0.05 vs. miR-384+Hoxa13-NC group, ^#^*P* < 0.05 vs. miR-384+Hoxa13 group

As shown in Fig. 5a, based on the prediction of the bioinformatics software (miRanda), we verified that Hoxa13 was a target of miR-384 by Dual-luciferase reporter assay. The luciferase activity was significantly decreased in the Hoxa13-Wt+miR-384 groups compared with Hoxa13-Wt+miR-384-NC group (Fig. 5a). Overexpression of Hoxa13 reversed miR-384-mediated exacerbation of CCI-induced neuronal apoptosis. Apoptosis rate of CCI-induced HT22 cells was attenuated in miR-384+Hoxa13 groups (Fig. 5b); CCI-induced apoptosis was also attenuated in miR-384+Hoxa13- (non-3′UTR) (Fig. 5b). Furthermore, we found that expression of FAM3A protein increased in miR-384+Hoxa13 groups (Fig. 5c) and also increased in miR-384+Hoxa13 (non-3′UTR) groups (Fig. 5c).

### Overexpression of Snhg8 combined with knockdown of miR-384 protected against CCI-induced neuronal apoptosis in vivo

**Fig. 6:**
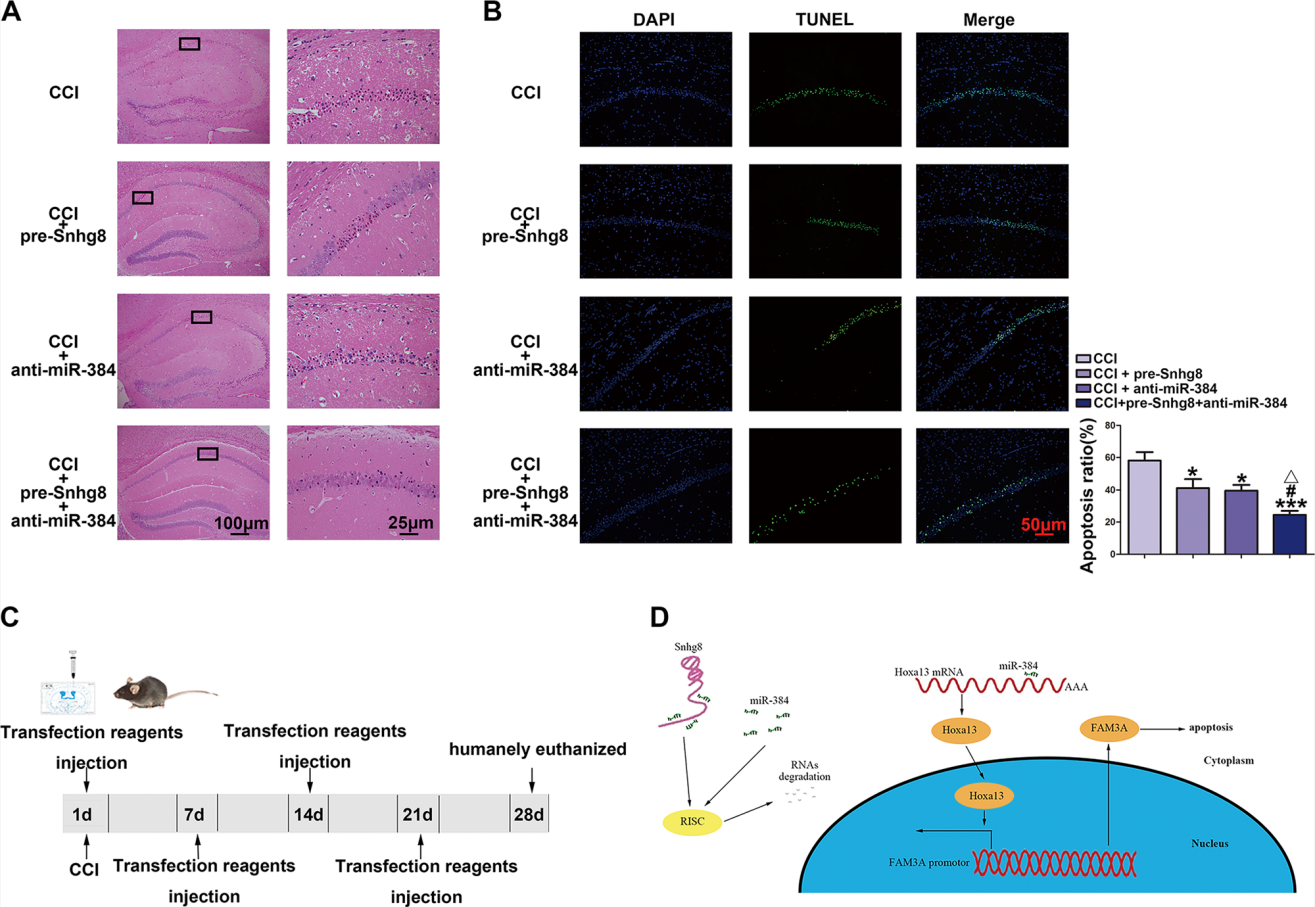
Overexpression of Snhg8 combined with knockdown of miR-384 protected against chronic cerebral ischemia (CCI) induced neuronal apoptosis in vivo. **a** H&E-stained (×100, ×400, scale bar = 100 and 25 μm) and **b** terminal deoxynucleotidyl transferase (TdT)-mediated dUTP-biotin nick end labeling (TUNEL) assay (×200, scale bar = 50 μm) 0f CA1 area of hippocampus tansfected with Snhg8 and miR-384 in vivo (*n* = 5, each group). Data are presented as mean ± SD. *P < 0.05 vs. CCI group, ***P < 0.001 vs. CCI group, ^#^*P* < 0.05 vs. CCI+pre-Snhg8, △*P* < 0.05 vs. CCI+miR-384 group. **c** Experimental schedule to explore the effects of Snhg8 and miR-384 on CCI-induced neuronal damage in vivo. **d** schematic illustration of the mechanism of Snhg8/miR-384/ Hoxa13/FAM3A axis regulating neuronal apoptosis

Given that Snhg8 functions as a ceRNA to regulate neuronal apoptosis by competing with Hoxa13 mRNA for binding to miR-384, we hope to further explore the significance of the association. So we injected pre-Snhg8, anti-miR-384 with in vivo transfection reagent into the lateral ventricle of mice in CCI mice model. Figure 6a is H&E-stained sections of the hippocampus CA1 region of each groups (×100 or ×400, scale bar = 100 or 25 μm). Figure 6b shows the effect of Snhg8 and miR-384 overexpression on CCI-induced neuronal apoptosis in hippocampus CA1 region (×200, scale bar = 50 μm). Apoptosis ratio of neurons decreased in CCI+pre-Snhg8 group (apoptotic rate = TUNEL-positive cell/total cell number), CCI+anti-miR-384 group and CCI+pre-Snhg8+anti-miR-384 group; compared with the CCI+pre-Snhg8 group, apoptosis ratio of neurons was decreased in CCI+pre-Snhg8+anti-miR-384 group; compared with the CCI+anti-miR-384 group, apoptosis ratio of neurons was decreased in CCI+pre-Snhg8+anti-miR-384 group. Figure 6c is the experimental schedule to explore the effect of Snhg8 and miR-384 on CCI-induced neuronal damage in vivo. Figure 6d is schematic illustration of the mechanism of Snhg8/miR-384/Hoxa13/FAM3A axis regulating neuronal apoptosis.

## Discussion

Extensive researches have been done to confirm the cerebral ischemia-related lncRNAs and the molecular mechanisms by which these lncRNAs regulate the development of ischemic cerebrovascular disease^20^. LncRNA regulates neuronal apoptosis seen in several studies^21^, for example, overexpression of lncRNA MALAT1 can inhibit glaucoma-induced neuron apoptosis through activation of PI3K/Akt-signaling pathway^22^; overexpression of lncRNA Rpa promote lead-induced neuronal apoptosis through PI3K/Akt-signaling pathway^23^. In the present study, we found that Snhg8 was lowly expressed in CCI-induced hippocampal neurons. Overexpression of Snhg8 inhibited CCI-induced apoptosis of hippocampal neurons, which demonstrated that Snhg8 exerted anti-apoptosis function in process of CCI-induced hippocampal neuronal apoptosis.

Through further research, we found that miR-384 was highly expressed in CCI-induced hippocampal neurons. And miR-384 acts as a pro-apoptosis gene in the process of CCI-induced hippocampal neuronal apoptosis. The results of this study were consistent with earlier research, in which miR-384 regulates the expression of PIWIL4 and then influence expression of apoptosis-related proteins, thus exerting pro-apoptotic effects in glioma cells^24^. Bioinformatics database (miRDB) showed that a putative-binding site existed between Snhg8 and miR-384. Therefore, we further verified that miR-384 targeted Snhg8-Wt by luciferase reporter assay. The RIP assay showed that Snhg8 and miR-384 were presented in a RISC complex. Overexpression of miR-384 downregulated the expression of Snhg8 and reversed the Snhg8-induced anti-apoptosis effect in CCI-induced hippocampal neurons. Also, overexpression of Hoxa13 decreased miR-384 expression. These results suggested that Snhg8 regulated CCI-induced neuronal apoptosis by targeting miR-384. Accumulated evidences have confirmed that lncRNAs bind with miRNAs and negatively regulate the expression of miRNAs, which influences neuronal apoptosis induced by ischemia and hypoxia^25^. MEG3 exerted pro-apoptosis function via sponging miR-147 in ischemia and hypoxia-induced neurons through NF-κB and Wnt/β signaling pathways^26^.

Our data confirmed that Hoxa13 is downregulated in CCI-induced hippocampal neurons and overexpression of Hoxa13 attenuated CCI-induced hippocampal neuronal apoptosis, which demonstrated the anti-apoptotic effect of Hoxa13. Studies have showed that Hoxa13 regulates tumor progression and embryonic limb formation through regulating cell apoptosis. For instance, Hoxa13 mutations in mice inhibit the apoptosis of urothelial cells by downregulating Bmp7 expression^27^; Hoxa13 is upregulated in lung cancer cells, and Hoxa13 knockdown promotes apoptosis of lung cancer cells^28^. On the basis that Hoxa13 had a putative binding site of miR-384, luciferase reporter assay confirmed that miR-384 bound to the Hoxa13 3´-UTR. Combined with the result that overexpression of miR-384 inhibited the expression of Hoxa13 protein, we thought that miR-384 negatively regulated the transcriptional expression of Hoxa13. Accumulated evidence indicated that miR-384 negatively regulates the expression of target gene by acting on its 3′UTR sequence: miR-384 negatively regulates the expression of Herpud1 by targeting its 3′UTR sequence and then regulates angiotensin II-induced vascular endothelial cell apoptosis^29^; miR-384 acts on IRS1 mRNA 3′UTR to inhibit its transcription, thereby inhibiting proliferation of human hepatoma cells^30^. Our study further found that overexpression of Snhg8 upregulated Hoxa13 protein expression and over-expression of miR-384 reversed Snhg8-induced promotion of Hoxa13 expression. Furthermore, both the 471–478 base sequence of Snhg8 and the 1630–1637 base sequence of Hoxa13 mRNA 3′UTR bind to the seed region of miR-384, and the seed region sequence is GAUCCUU, that is, the MRE exists. These results strongly suggested that Snhg8 acted as a ceRNA of miR-384 and regulated the expression of Hoxa13. Studies have revealed that LncRNAs can serve as ceRNAs for certain miRNAs and can modulate neuronal apoptosis by competing with downstream genes for miRNA-binding sites^31^. For example, Long non-coding RNA MEG3 functions as a competing endogenous RNA to regulate ischemic neuronal death by targeting miR-21/PDCD4-signaling pathway^32^; LncRNA H19 acts as a ceRNA regulating miR-let-7a/NGF-signaling pathways to regulate neuronal apoptosis induced by subarachnoid hemorrhage^33^.

Our results showed FAM3A was downregulated in CCI-induced hippocampal neurons and overexpression of FAM3A inhibited CCI-induced hippocampal neuronal apoptosis. FAM3A protects HT22 cells against hydrogen peroxide-Induced oxidative stress through activation of PI3K/Akt12 pathway^34^. Silico analysis (JASPAR) suggested that Hoxa13 might bind to the promoter sequence of FAM3A. ChIP assays corroborate our hypothesis that Hoxa13 could directly bind to FAM3A promoter. In view of the result that overexpression of Hoxa13 upregulated the expression of FAM3A mRNA and protein, indicated that Hoxa13 positively regulated the transcriptional expression of FAM3A. Consistent with the results of this study, the reports that Hoxa13 exerts positive transcriptional regulation are as follows: Wu et al. find that Hoxa13 promotes the transcriptional expression by targeting the BMP7 promoter region and acts as a oncogene in gastric cancer^35^. Hoxa13 targets the Aldh1a2 promoter and up-regulate its expression, regulating the limb morphogenesis^18^. Through further research, we found that overexpression of Hoxa13 upregulated the expression of FAM3A protein, and suppress CCI-induced hippocampal neuronal apoptosis. Moreover, the rescue experiment showed that overexpression of miR-384 reversed Hoxa13-induced promotion of FAM3A and anti-apoptosis effect by binding with Hoxa13 3′-UTR. Above results dramatically revealed that miR-384 could negatively regulate the expression of Hoxa13, further affect the transcriptional regulation of Hoxa13 to FAM3A and regulate the CCI-induced hippocampal neuronal apoptosis.

In general, we demonstrated that the Snhg8/miR-384/Hoxa13/FAM3A axis played a vital role in CCI-induced hippocampal neuronal apoptosis (Fig. 6d). Finally, we proved that the overexpression of Snhg8 combined with knockdown of miR-384 exhibited the lowest neuron apoptosis ratio and the highest percent survival in vivo. This study might provide a new mechanistic insight into the pathogenesis of CCI and identify novel targets for the treatment.

## Materials and methods

### HT22 cell culture, CCI treatment

HT22, the mouse hippocampal neuronal cell line, was purchased from ATCC (Gaithersburg, MD, USA). The cells were cultured in DMEM medium containing 10% fetal bovine serum and antibiotics (100 μg/ml streptomycin, 100 U/ml penicillin) at 37 °C in a humidity chamber with 5% CO_2_ and 95% air. HT22 cells were exposed to CCI treatment as previously described^36^. In brief, the culture medium was replaced with glucose-free medium for 48 h at 37 °C with a humidified atmosphere of 3% O_2_ and 5% CO_2_ in a tri-gas incubator (SANYO, Osaka, Japan). As controls, the culture medium was replaced with fresh DMEM medium for 48 h at 37 °C with a humidified atmosphere of 95% air and 5% CO_2_. To identify the effect of CCI on HT22 cells, the cells were divided into two groups: Control group and CCI group.

### Animals and experimental design

Adult (12–14 weeks of age) male C57BL/6J mice were used for CCI operation. The use and care of animals were performed in accordance with the guidelines for the Care and Use of Department of Laboratory Animal Science of China Medical University and approved by the Institutional Animal Care and Use Committee. ARRIVE guidelines were followed in the preparation of the manuscript. Mice were housed individually in a regulated environment of humidity and temperature (12 h light/dark cycle, lights on at 08:00) with standard mouse diet and water. To identify the effect of CCI on hippocampal neurons, the mice weighing 24–29 g were randomly assigned to two groups: Sham group (*n* = 14) and CCI group (*n* = 30); to identify the effect of Snhg8 and miR-384 on hippocampal neuronal apoptosis, the mice weighing 24–29 g were randomly assigned to four groups: CCI group (*n* = 30), CCI+pre-Snhg8 group (*n* = 25), CCI+anti-miR-384 group (*n* = 25), and CCI+pre-Snhg8+anti-miR-384 group (*n* = 20).

### Surgical procedure and entransfer

Mice were anesthetized with 1.5% isoflurane. And operation was conducted after confirming the mice being completely static and unresponsive to a toe pinch. Both CCAs were exposed through midline cervical incision and freed from their sheaths. In the CCI group, the ACs (Research Instruments SW, Escondido, USA) were applied to the bilateral CCAs; Sham procedures were performed in the same fashion without implantation of ACs. Rectal temperature was maintained between 36.5 and 37.5 °C. CCI-induced mice received intracerebrao-ventricular (IVC) injections of Snhg8 full-length plasmid and plasmid vector, miR-384 antagomir (anti-miR-384) using Entranster in vivo DNA/RNA reagent (Engreen Biosystem Co., Ltd., China). IVC injections of plasmid were executed during the CCI operation and every 7 days after CCI operation at the quantity of 2.5 μg/10 μl per mouse, which was to maintain the effect of entransfer^37^.

### Quantitative real-time polymerase chain reaction (PCR)

Total RNA was extracted from CA1 region of hippocampal (Fig. 1b), or cultured HT22 cells (Figs. 1d, 2a, 3a, 4a, 4b, 5a, 6a and 7A) by using TRIzol reagent (Life Technologies Corporation, Carlsbad, CA, USA). RNA concentration and quality of each sample were determined with Nanodrop Spectrophotometer (ND-100) by the 260/280 nm ratio. The primers for Snhg8, Hoxa13, and β-actin were synthesized from Takara Bio Inc. The expression levels of miR-384 and U6 (Applied Biosystems, Foster City, CA, USA) were examined with High Capacity cDNA Reverse Transcription Kits (Applied Biosystems, Foster City, CA, USA) and Taqman Universal Master Mix II (Life Technologies Corporation, Carlsbad, CA, USA).

Snhg8: forward 5′-GACACAAGGTGGCTATGGTGCTG-3′,

reverse 5′-CATGGTGGTCGTCGCGCTAAC-3′;

Hoxa13: forward5′-TACTTCGGCAGCGGCTACTACC-3′,

reverse 5′-CGGCGGTGTCCATGTACTTGTC-3′;

FAM3A: forward 5′-TTGGCCTTCCTCGAATTCAGCAG-3′,

reverse 5′-GCTCAGGTGCTCTTCAGGACAAG-3′;

β-actin: forward 5′-GTGACGTTGACATCCGTAAAGA-3′,

reverse 5′-GTAACAGTCCGCCTAGAAGCAC-3′.

The expression levels of miR-384 and U6 (Applied Biosystems, Foster City, CA, USA) were examined with High Capacity cDNA Reverse Transcription Kits (Applied Biosystems, Foster City, CA, USA) and Taqman UniversalMaster Mix II (Life Technologies Corporation, Carlsbad, CA, USA). The relative quantification 2^−△△Ct^ method was applied to calculate the gene expression.

### Apoptosis analysis

HT22 cell apoptosis was evaluated by Annexin V: FITC Apoptosis Detection Kit I (Southern Biotech, Birmingham, AL, USA). After washing with 4 °C PBS twice, cells were collected and stained with Annexin V-FITC according to the manufacturer’s instructions. Then the cells were analyzed by flow cytometry (FACScan, BD Biosciences) and apoptotic fractions were investigated by CELL Quest 3.0 software (FACScan, BD Biosciences). Neuronal apoptosis of hippocampal CA1 region was evaluated by the terminal deoxynucleotidyl transferase (TdT)-mediated dUTP-biotin nick end labeling (TUNEL) assay according to the manufacturer’s protocol. Briefly, sections were deparaffinized and washed three times with PBS for 5 min, then pre-treated with proteinase K (20 mg/ml) in Tris–hydrochloride buffer for 30 min at room temperature. After three 5-min washes with PBS, sections were incubated with 50 μl of TUNEL reaction mixture for 60 min in a humidified chamber at 37 ℃. Sections were counterstained with 4′,6-diamidino-2-phenylindole (DAPI; KeyGen Biotech, Nanjing, China). As a negative control, TdT was replaced with distilled water in the working solution. Apoptotic cells showed green fluorescence when visualized under a fluorescence microscope (Olympus IX71; Olympus, Tokyo, Japan) equipped with a fluorescein filter (520 nm). DAPI-stained nucleus were visible as a blue fluorescent signal when visualized using a 365-nm filter. TUNEL-positive cells in the hippocampal CA1 region were counted at × 200 magnifications. Six randomly selected fields were counted for each sample and are expressed as the ratio of the number of apoptotic to total number of neurons of at least three independent experiments.

### RNA immunoprecipitation (RIP)

HT22 cells were lysed by a complete RNA lysis buffer with protease inhibitor and RNase inhibitor from an EZ-Magna RIP kit (Millipore, MA, USA) according to the manufacturer’s protocol. Whole cell lysate of the control groups and anti-miR-384 groups were incubated with RIP immunoprecipitation buffer containing magnetic beads conjugated with mouse anti-Argonaute2 (Ago2) antibody (Millipore, MA, USA), and NC normal mouse IgG (Millipore, MA, USA). Samples were incubated with Proteinase K buffer and then immunoprecipitated RNA was isolated. The RNA concentration was measured by a NanoDrop (Thermo Scientific, Waltham, MA, USA) and the RNA quality assessed using a bioanalyser (Agilent, Santa Clara, CA, USA). Furthermore, purified RNA was obtained and analyzed by qRT-PCR to demonstrate the presence of the binding targets using respective primers mentioned earlier.

### Western blot analysis

Total proteins from the cells or tissues were extracted by RIPA buffer with protease inhibitors (Beyotime Institute of Biotechnology, Jiangsu, China) on ice. Electrophoresis was conducted to equal amount of protein samples (40 mg) with sodium dodecyl-sulfate–polyacrylamide gel electrophoresis (SDS–PAGE) and then transferred to PVDF membranes. Membranes were incubated in 5% non-fat milk dissolved in Tris-buffered saline (TBS) containing 0.1% Tween-20 for 2 h at room temperature and then incubated with primary antibodies against Hoxa13 (1:1000, ABcam, Santiago, USA), FAM3A (1:1000, ABclonal, Boston, USA), and β-actin (1:1000, Proteintech, Chicago, IL) at 4 ℃ overnight. On next day, membranes were incubated with secondary antibodies (goat anti-rabbit or goat anti-mouse, 1:10,000, respectively; Proteintech, Chicago, IL) at room temperature for 2 h. Immunoblots were visualized by enhanced chemiluminescence (Santa Cruz Biotechnology) and scanned using ChemImager 5500 V2.03 software. Then FluorChem 2.0 software was used to calculate the integrated density values (IDV).

### Chromatin immunoprecipitation assay (ChIP)

According to the manufacturer’s instructions, simple ChIP Enzymatic Chromatin IP Kit (Cell Signaling Technology, Danvers, MA, USA) was applied to ChIP assays. Briefly, cells were crosslinked with EBM-2 containing 1% formaldehyde and collected in lysis buffer. And then micrococcal nuclease was used to digest the chromatids. Immunoprecipitation was incubated with 3 mg of anti-Hoxa13 antibody (1:1000, ABcam, UK) or normal rabbit IgG followed by immunoprecipitating with Protein G Agarose Beads and stored at 4 ℃ overnight with gentle shaking. Then the DNA crosslink was reversed by 5 mol/l NaCl and Proteinase K. Finally, DNA was purified. Immunoprecipitated DNAs were amplified by PCR according to their specific primers as follows:

Control PCR1: forward 5′-TGAAAATGTGGACTAGAGCCAGA-3′;

reverse 5′-CAGACACTCCAGAACAGGGC-3′;

AGGF1PCR2: forward 5′-TCCCCGCATCCATCCTCTTA-3′;

reverse 5′-CCCATTCCCACTTCCTCCAC-3′.

### Reporter vectors construction and luciferase assay

Snhg8 full-length and Hoxa13 3′-UTR sequences were amplified by PCR and cloned into a pmirGlo Dual-luciferase miRNA Target Expression Vector (Promega, Madison, WI, USA) to construct luciferase reporter vector (Snhg8-Wt and Hoxa13-Wt, GenePharma). Mutate putative-binding site of Snhg8 or Hoxa13 replaced the sequence of putative-binding site (Snhg8-Mut and Hoxa13-Mut). HT22 cells were seeded in 96-well plates and the cells were cotransfected with Snhg8-Wt (or Snhg8-Mut) or Hoxa13-Wt (or Hoxa13-Mut) and miR-384 or miR-384-NC plasmids when they were at 50–70% confluence. After 48 h of transfection by Dual- Luciferasereporter assay kit (Promega), the luciferase activities were measured. The cells were divided into five groups, respectively: Control group, Snhg8-Wt+miR-384-NC (transfected with Snhg8-Wt and pre-miR-384-NC), Snhg8-Wt+miR-384 group (transfectedwith Snhg8-Wt and pre-miR-384), Snhg8-Mut+miR-384-NC group (transfected with Snhg8-Mut and pre-miR-384-NC), and Snhg8-Mut+miR-384 group (transfected with Snhg8-Mut and pre-miR-384); Control group, Hoxa13-Wt+miR-384-NC group (transfected with Hoxa13-Wt and pre-miR-384- NC), Hoxa13-Wt+miR-384 group (transfected withHoxa13-Wt and pre-miR-384), Hoxa13-Mut+miR-384-NC group (transfectedwith Hoxa13-Mut and pre-miR-384-NC), and Hoxa13-Mut+miR-384 group (transfected with Hoxa13-Mut and pre-miR-384).

### Cell transfection

PcDNA3.1-Snhg8 full-length plasmid (pre-Snhg8) and the nontargeting sequence (NC); miR-384 agomir (pre-miR-384), miR-384 antagomir (anti-miR-384), and their respective nontargeting sequence (pre-NC or anti-NC) were synthesized (GenePharma, Shanghai, China). Hoxa13 full length (with 3′-UTR) (Hoxa13 (+) or Hoxa13) plasmid, Hoxa13 (without 3′-UTR) (Hoxa13 (non-3′UTR)) plasmid, and their respective non-targeting sequences (negative control, NC) (Hoxa13-NC or sh-NC); FAM3A full length (FAM3A (+)) plasmid and the respective non-targeting sequences (negative control, NC) (FAM3A (+) -NC) were constructed (Life Technologies, Waltham, MA, USA). After seeded into 24-well plates (Corning), HT22 cells were transfected with the plasmids via Opti-MEM and Lipofectamine 3000 reagent (Life Technologies Corporation) according to the manufacturer’s instructions when they were at 50–70% confluence. Geneticin (G418; Sigma-Aldrich) was utilized to select the G418-resistant clones after 3–4 weeks.The overexpression and the silence efficiency were analyzed using qRT-PCR. To identify the effect of Snhg8 on CCI-induced hippocampal neuron apoptosis, cells were divided into three groups: Control group, pre-NC group (tansfected with pcDNA-NC plasmid), and pre-Snhg8 group (tansfected with Snhg8 full length plasmid). Likewise, to determine the effect of miR-384 on CCI-induced hippocampal neuron apoptosis, cells were divided into five groups: Control group, pre-NC group (transfected with NC), pre-miR-384 group (transfected with miR-384 agomir), anti-NC group (transfected with NC), and anti-miR-384 (transfected with miR-384 antagomir). To investigate the effect of Hoxa13 on CCI-induced hippocampal neuronal apoptosis, cells were divided into five groups: Control group, Hoxa13 (+)-NC group (transfected with empty plasmid), Hoxa13 (+) group (transfected with Hoxa13 full length plasmid. Furthermore, to verify the underlying mechanism of Snhg8 regulated the CCI-induced hippocampal neuronal apoptosis via decreasing miR-384, cells were divided into five groups: Control group, pre-NC+pre-NC group, Snhg8 (+)+miR-384 group (pcDNA-Snhg8 stable expressing cells cotransfected with pre-miR-384).

### Statistical analysis

Data are presented as mean ± standard deviation (SD). All statistical analyses were evaluated by SPSS 23.0 statistical software with the Student’s *t*-test or one-way analysis of variance ANOVA. Differences were considered to be significant when *P* < 0.05. Corresponding significance levels were indicated in the figure.

## Supplementary information

Fig.1C

Fig.2A

Fig.2C

Fig.3C

Fig.4A

Fig.4C

Fig.5B
