# Correction: Mechanism of Snhg8/miR-384/Hoxa13/FAM3A axis regulating neuronal apoptosis in ischemic mice model

**DOI:** 10.1038/s41419-019-1865-x

**Published:** 2019-08-21

**Authors:** Jie Liu, Ping An, Yixue Xue, Dongfang Che, Xiaobai Liu, Jian Zheng, Yunhui Liu, Chunqing Yang, Zhen Li, Bo Yu

**Affiliations:** 1 Department of Neurosurgery, Shengjing Hospital of China Medical University, Shenyang, China; 2 Key Laboratory of Neuro-oncology in Liaoning Province, Shenyang, China; 3 Liaoning Clinical Medical Research Center in Nervous System Disease, Shenyang, China; 4 Department of Neurobiology, College of Basic Medicine, China Medical University, Shenyang, China; 5 Key Laboratory of Cell Biology, Ministry of Public Health of China, China Medical University, Shenyang, China; 6 Key Laboratory of Medical Cell Biology, Ministry of Education of China, China Medical University, 110122 Shenyang, China

**Keywords:** Long non-coding RNAs, miRNAs, Cell death in the nervous system

**Correction to:** **Cell Death and Disease**

10.1038/s41419-019-1631-0 Published online 5 June 2019

After publication of the article, we realized that a Natural Science Foundation of China grant (81571868) was omitted in the acknowledgements. The correct Acknowledgements are listed below:

This work is supported by grants from the Natural Science Foundation of China (81571868, 81672511 and 81573010), Liaoning Science and Technology Plan Project (No. 2017225020), Liaoning Science and Technology Plan Project (No. 2015020475), Project of Key Laboratory of Neuro-oncology in Liaoning Province (112-2400017005), special developmental project guided by central government of Liaoning Province (No. 2017011553-301).

This has been corrected in both the PDF and HTML versions of the article.

This correction does not impact the results and conclusions of the article.

