# Supplementary figures and images for: Mechanism of Snhg8/miR-384/Hoxa13/FAM3A axis regulating neuronal apoptosis in ischemic mice model
Source: Cell Death Dis. 2019 Jun 5;10(6):441. doi: 10.1038/s41419-019-1631-0 (PMC6549185; doi:10.1038/s41419-019-1631-0)

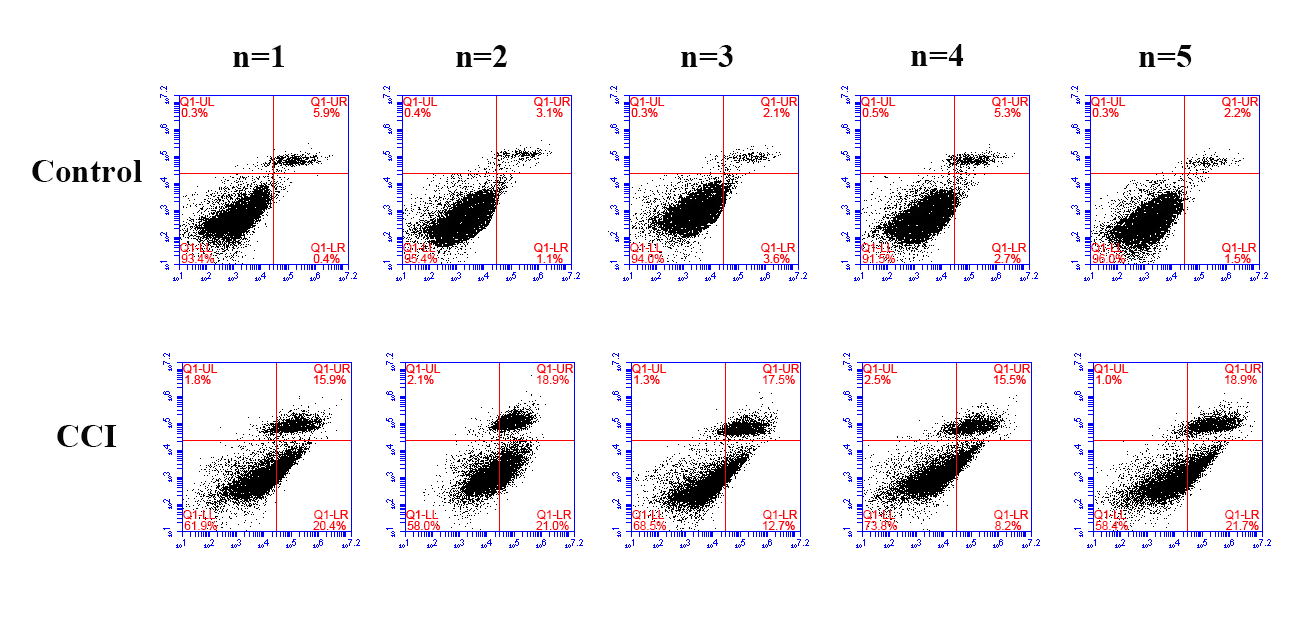

Supplement: Supplementary file 1 — Fig.1C [file 41419_2019_1631_MOESM1_ESM.tif]

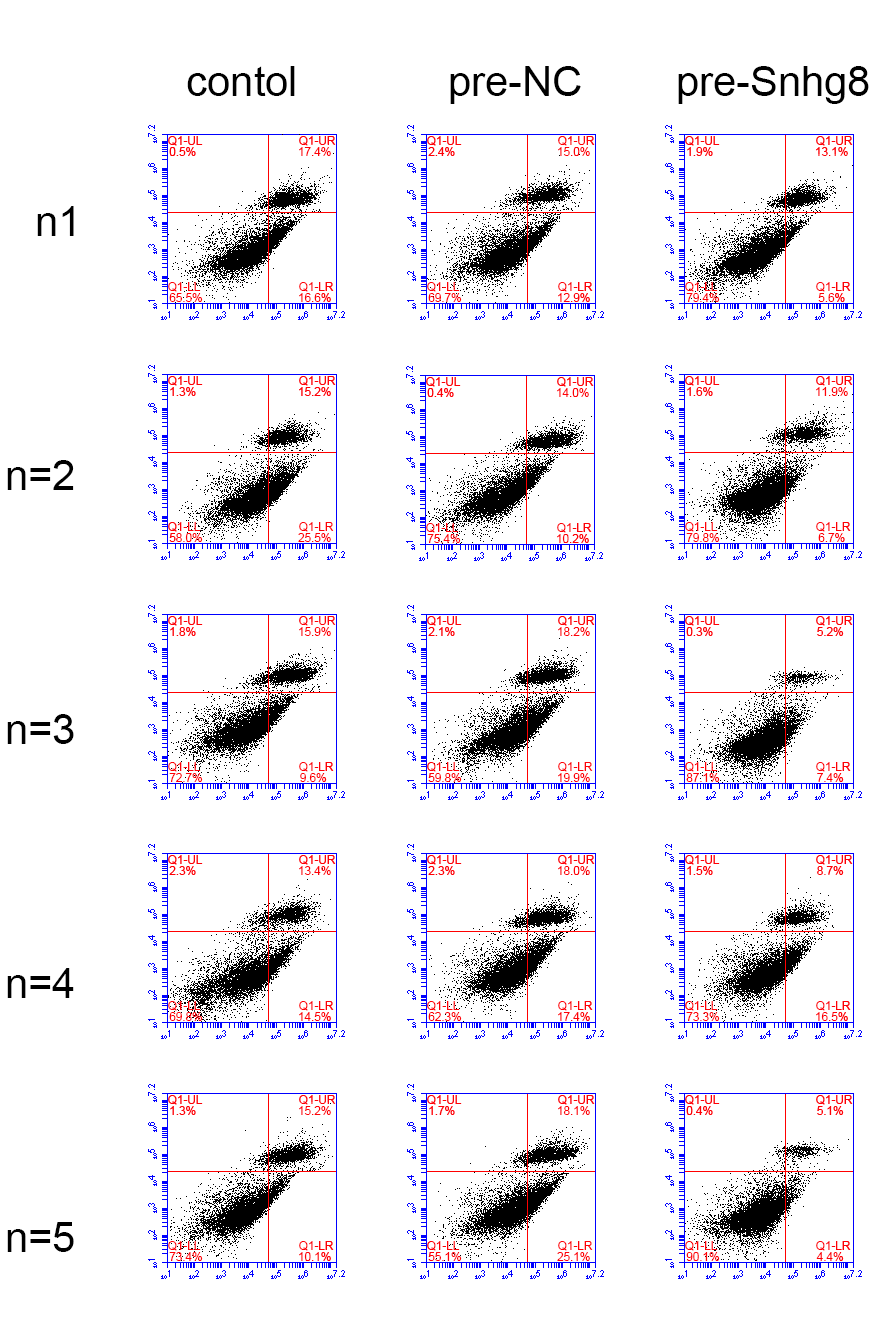

Supplement: Supplementary file 2 — Fig.2A [file 41419_2019_1631_MOESM2_ESM.tif]

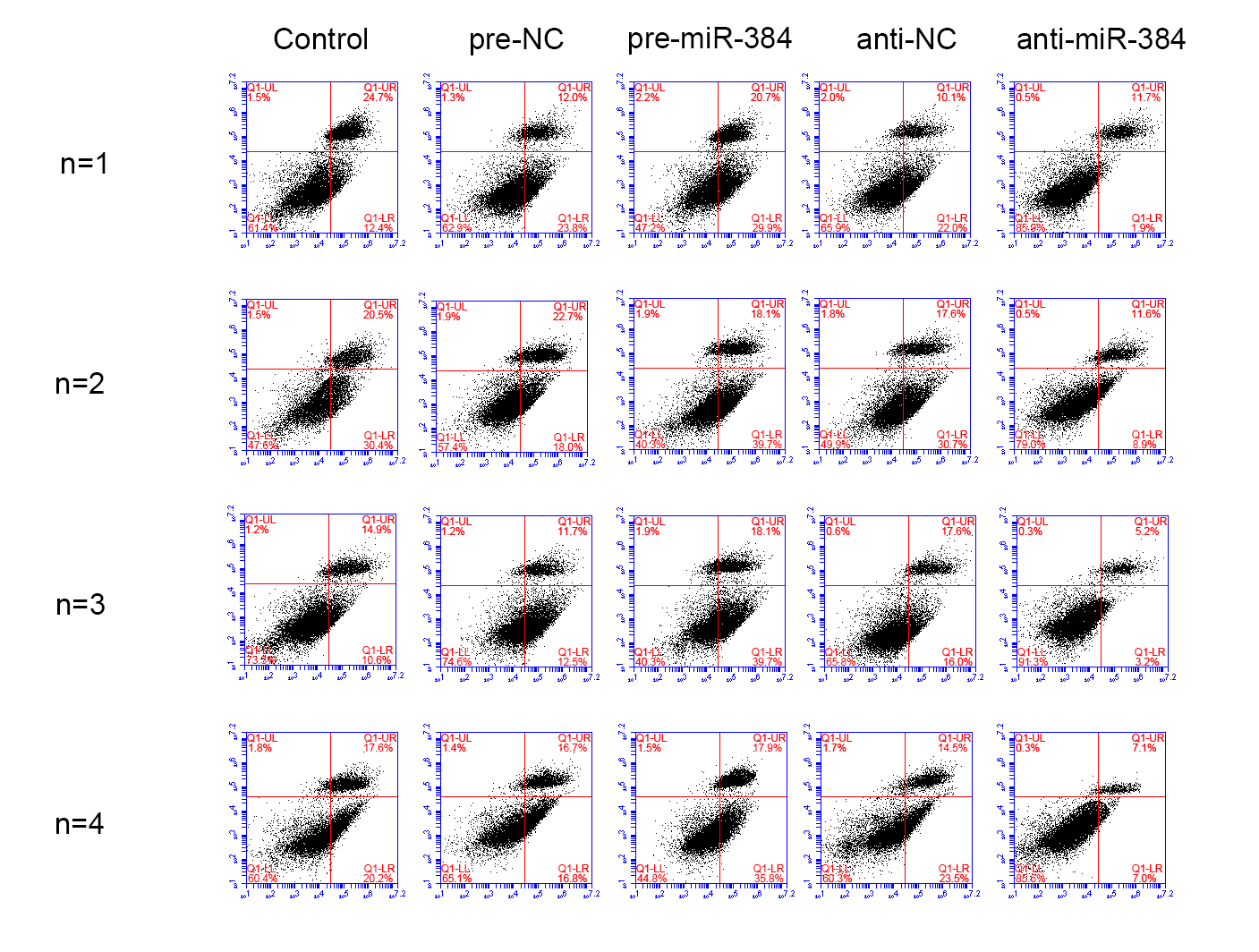

Supplement: Supplementary file 3 — Fig.2C [file 41419_2019_1631_MOESM3_ESM.tif]

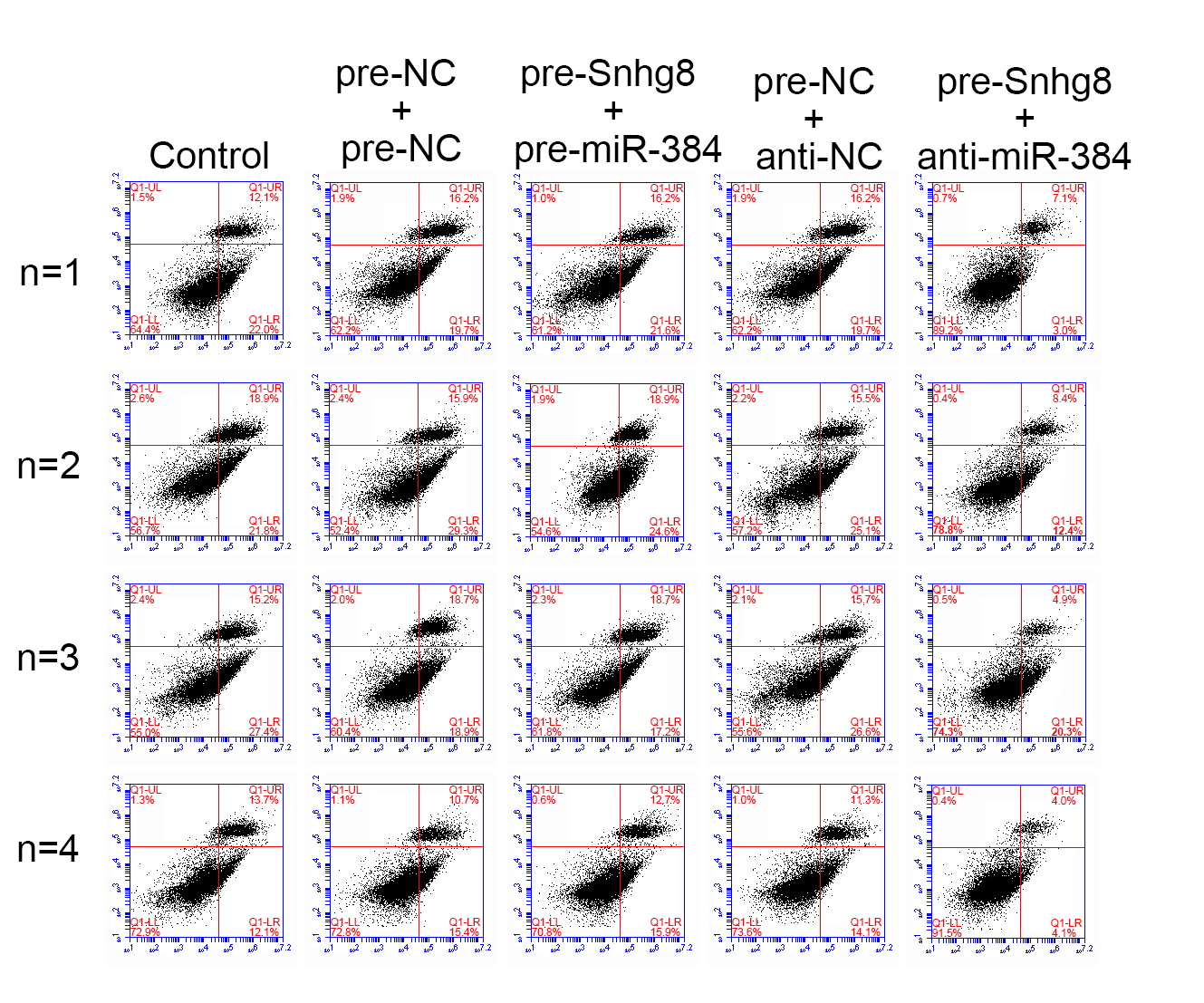

Supplement: Supplementary file 4 — Fig.3C [file 41419_2019_1631_MOESM4_ESM.tif]

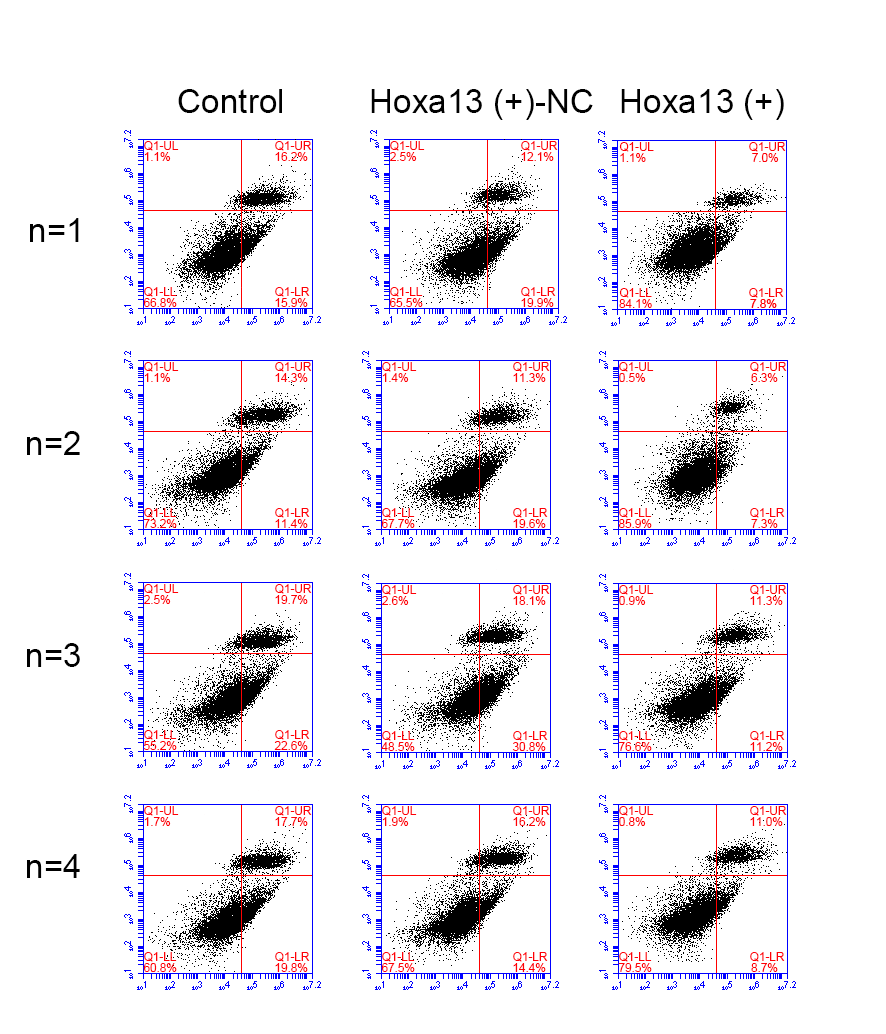

Supplement: Supplementary file 5 — Fig.4A [file 41419_2019_1631_MOESM5_ESM.tif]

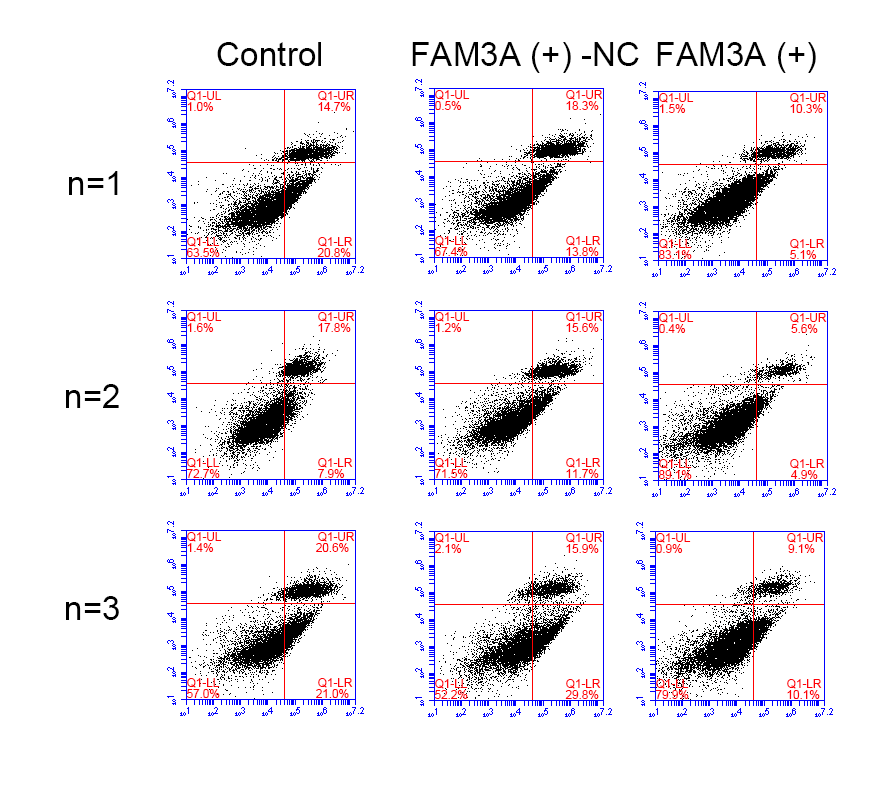

Supplement: Supplementary file 6 — Fig.4C [file 41419_2019_1631_MOESM6_ESM.tif]

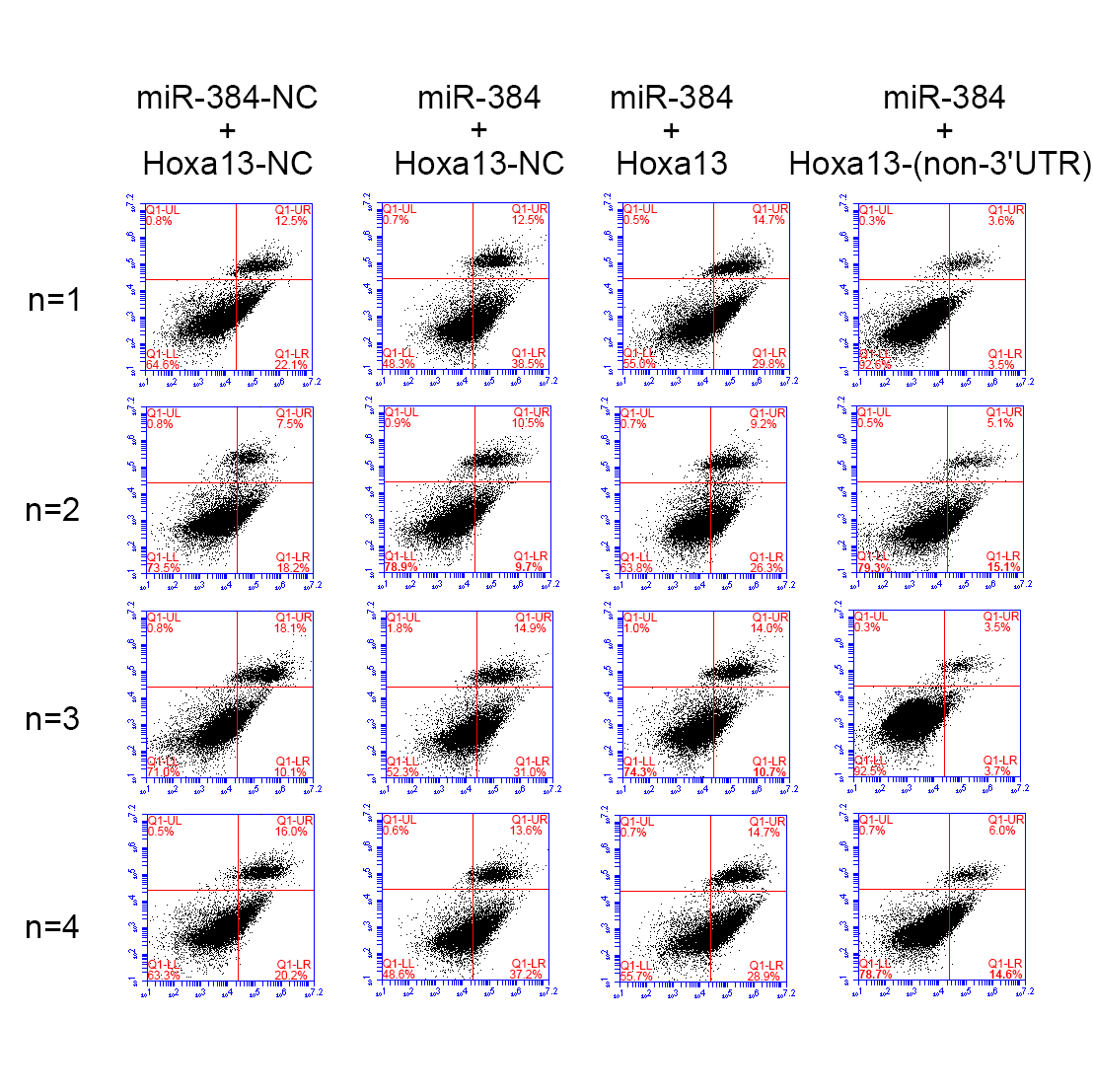

Supplement: Supplementary file 7 — Fig.5B [file 41419_2019_1631_MOESM7_ESM.tif]
